# 

**Published:** 1889-09

**Authors:** 


CONVENIENT METHODS OF MEDICATION.—On this important subject read the advertisement to be found on the last cover page of this Journal.

WOOD'S MEDICAL AND SURGICAL MONOGRAPHS, published monthly, at 10cts. per copy or $1.00 per annum.

The number before us is for July, and is neatly gotten out with stiff paper back. It contains 253 large oc. pages, and is filled with the following papers ably presented:

Cancer and Cancerous Diseases, by Sir Spencer Wells, Bart., F. R. C. S.; The influence of menstruation, and of the pathological condition of the uterus on cutaneous diseases, by Dr. L. Grellety; Tension as met with in surgical practice; Inflammation of bone, cranial and intracranial injuries, by T. Bryant, F. R. C. S.; Antisepsis and its relation to bacteriology, by Dr. J. Neudorpher.

The Treatment of Strictures of the Urethra by Electrolysis, by S. M. Hogan, M. D., of Union Springs, Ala. Reprinted from Tranactions of Southern Surgical and Gynecological Association, 1888. This is an able and interesting paper, containing reports of cases treated, with valuable and practical suggestions in relation to this new method of treatment.

RECEIPTED FOR 1889.—W J McAlpine, H Allison, to July; E A Speed, S W Earle, J Smith, R A Boyan, H H Phillips, R D Jons.

See Special Notice of College of Physicians and Surgeons, Baltimore.

THE TENTH INTERNATIONAL MEDICAL CONGRESS will be held in Berlin, and it is proposed that the sessions begin Aug. 6, 1890.

THE AMERICAN RHINOLOGICAL ASSOCIATION will hold its seventh annual meeting at Chicago, Ill., Oct. 9, 10 and 11, 1889.

THE MISSISSIPPI VALLEY MEDICAL ASSOCIATION will hold its next annual meeting at Evansville, Ind., Sept. 24, 25 and 26, 1889.

